# Supplementary material for: Fat Body Cells Are Motile and Actively Migrate to Wounds to Drive Repair and Prevent Infection
Source: Dev Cell. 2018 Feb 26;44(4):460–470.e3. doi: 10.1016/j.devcel.2018.01.026 (PMC6113741; doi:10.1016/j.devcel.2018.01.026)
Supplement: Document S1. Figures S1–S4 and Table S1 [file mmc1.pdf]

Developmental Cell, Volume *44*

Supplemental Information

Fat Body Cells Are Motile and Actively Migrate to Wounds to Drive Repair and Prevent Infection

Anna Franz, Will Wood, and Paul Martin

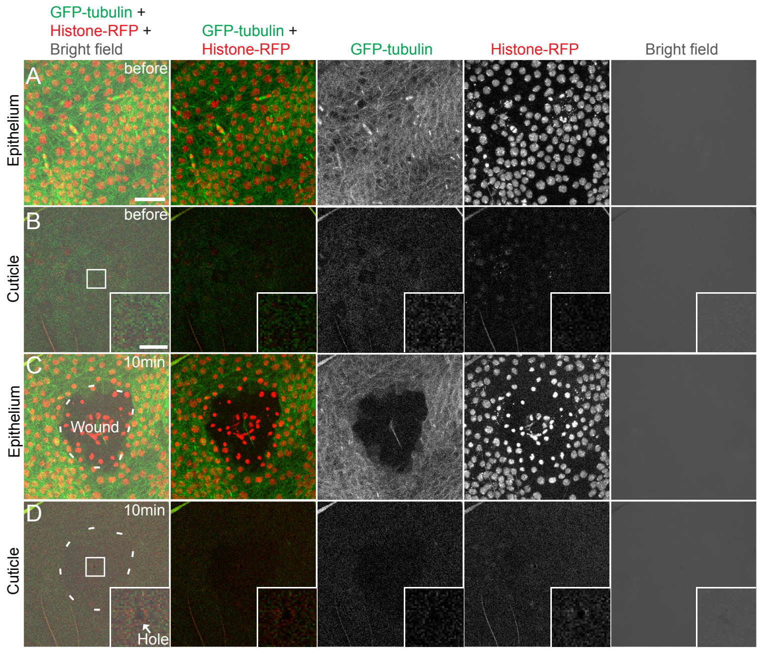

Figure S1

Morphology of a laser-induced epithelial wound labelled with Histone-RFP. Related to Figure 1

(A-D) Microscopy images of a Z-stack projection restricted to the epithelium (A, C) or the cuticle (B, D) before (A, B) and 10 minutes after (C, D) making a laser-induced wound (outlined by white dotted line) in a c564-Gal4+Ubi>GFP-tubulin+Ubq>Histone-RFP pupa (Tubulin in green; nuclei in red; bright field in grey; magnified in the insets to show the hole in the cuticle).

Scale bars=20μm and 5μm (insets).

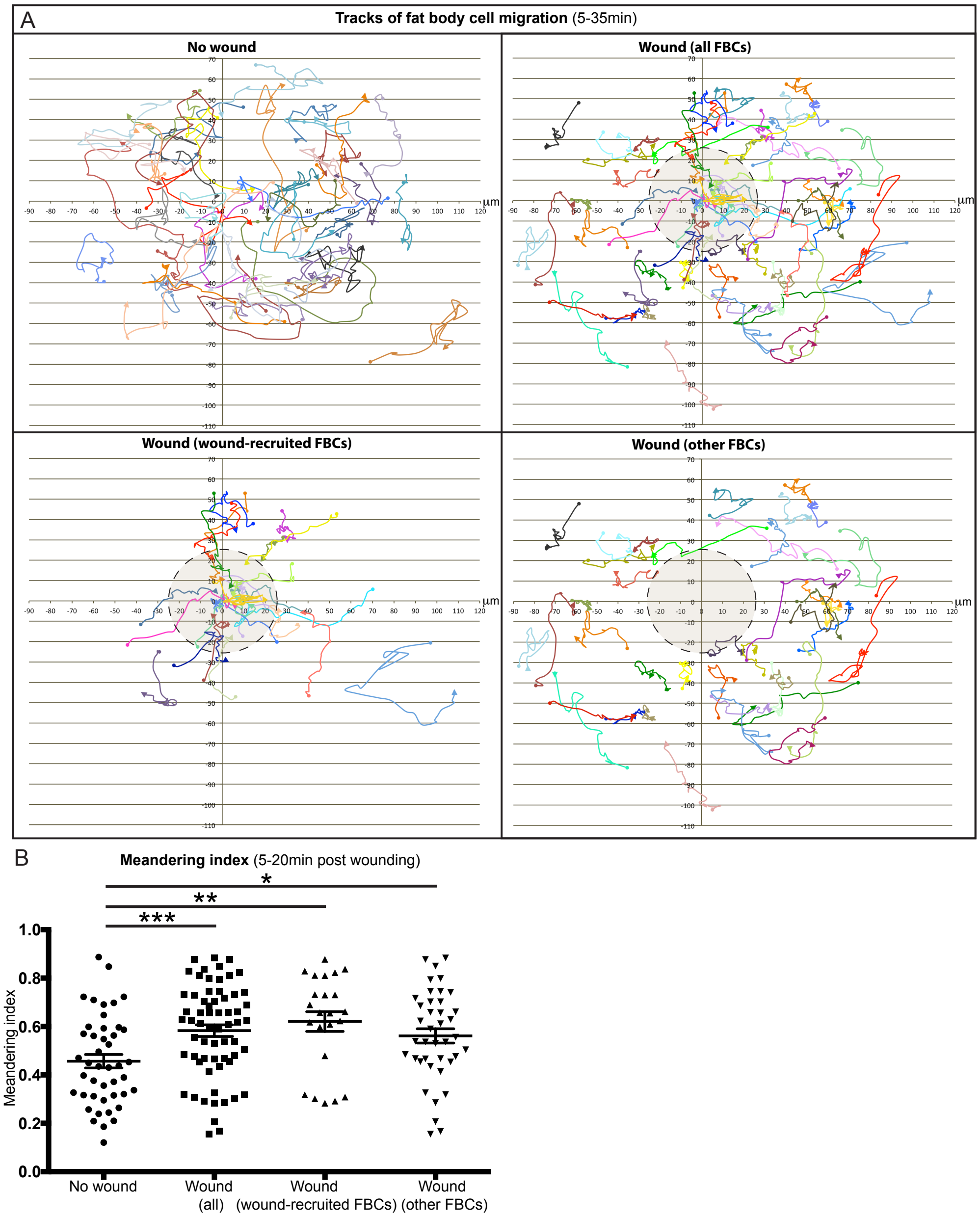

Figure S2

Fat body cells migrate to wounds with a high meandering index. Related to Figure 2

(A-B) Migration tracks of FBCs (A; 5-35min) and quantification of meandering index (B, 5-20min) of FBC migration in c564-Gal4+UAS-GFP+Ubq>Histone-RFP unwounded (n=44 cells) or wounded pupae (tracks from all FBCs displayed on top right; from wound-recruited FBCs displayed on bottom left; and from FBCs that do not to interact with the wound area, displayed on bottom right; n=63, 23 and 40 cells, respectively; tracking performed in Z-projection mode). Mean±SEM. * $P<0.05$, ** $P<0.01$, *** $P<0.001$ (Student's T-Test)

.

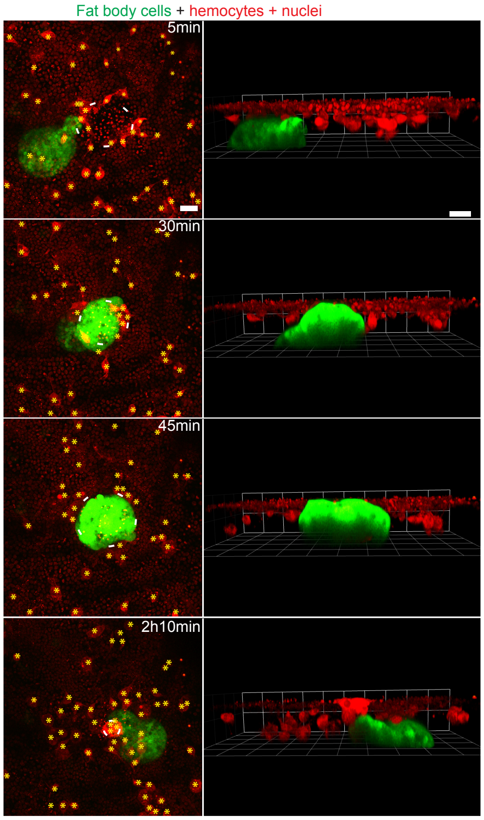

Figure S3
Migration of a fat body cell and hemocytes to a wound.
Related to Figure 4

Still images from a timelapse movie showing hemocyte and FBC recruitment to a wound in a c564-Gal4+UAS-GFP+srp>mCherry+Ubq>Histone-RFP pupa (projection (left) and Z-plane (right) views; FBCs in green; hemocytes in red with yellow asterisks; epithelial nuclei in red; wound area containing bright red nuclei + outlined with white dotted line). Elapsed time is in top right corner. Scale bars=20µm. See also Movie S8.

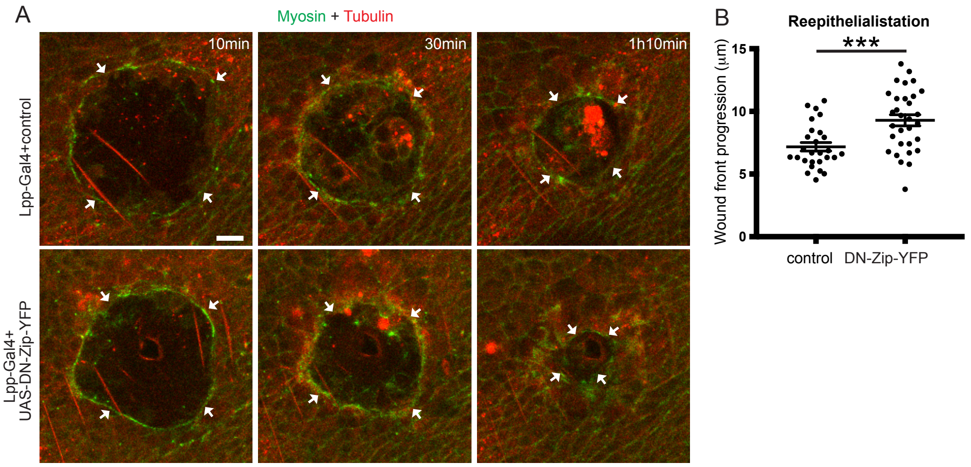

Figure S4

Reepithelialisation is faster in the absence of fat body cells at wounds. Related to Figure 3

(A-B) Still images from a timelapse of reepithelialisation (A) and quantification of wound front progression (30-70min, B) in sqh>Sqh-GF-P+Ubq>RFP-tub+Lpp-Gal4+control or +UAS-DN-Zip-YFP pupae (n=27 and 31, respectively; Myosin in green; Tubulin in red; wounds margins indicated with arrows). Mean±SEM. ***P<0.001 (Student's T-Test). Control=w67. Scale bar = 10μm.

Table S1

Genotypes of pupae used in each experiment.

Related to Figures 1-5 and Supplementary Figures 1-4.

Figure	Genotype
Fig 1A	w67
Fig 1C-I	c564-Gal4+UAS-GFP+Ubq>Histone-RFP
Fig 2A-D	c564-Gal4+UAS-GFP+Ubq>Histone-RFP
Fig 3A and C	Lpp-Gal4+UAS-GMA
Fig 3D and E	Lpp-Gal4+UAS-rd-Tomato+w67 and<br>Lpp-Gal4+UAS-rd-Tomato+UAS-DN-Zip-YFP
Fig 4A	srp-Gal4+c564-Gal4+UAS-GFP+UAS-Red- Stinger
Fig 4B and C	srp-Gal4+UAS-Reaper+tubGal80ts+srp-GMA+Ubq>Histone-<br>RFP
Fig 4D	c564-Gal4+UAS-GFP+Ubq>Histone-RFP
Fig 5A	c564-Gal4+UAS-GFP+Ubq>Histone-RFP
Fig 5B and C	w67
Fig 5D	c564-Gal4+UAS-GFP-Fascin+Ubq>RFP-tubulin
Figure 5 E	c564-Gal4+UAS-GFP+Ubq>Histone-RFP
Figure 5 F	Attacin>GFP+Lpp-Gal4+UAS-myr-td-Tomato
Fig S1A-D	c564-Gal4+Ubi>GFP-tubulin+Ubq>Histone-RFP
Fig S2A and B	c564-Gal4+UAS-GFP+Ubq>Histone-RFP
Fig S3	c564-Gal4+UAS-GFP+srp>mCherry+Ubq>Histone-RFP
Fig S4 A and B	sqh>Sqh-GFP+Ubq>RFP-tub+Lpp-Gal4+w67 and<br>sqh>Sqh-GFP+Ubq>RFP-tub+Lpp-Gal4+UAS-DN-Zip-YFP
