# Supplementary material for: Fat Body Cells Are Motile and Actively Migrate to Wounds to Drive Repair and Prevent Infection
Source: Dev Cell. 2018 Feb 26;44(4):460–470.e3. doi: 10.1016/j.devcel.2018.01.026 (PMC6113741; doi:10.1016/j.devcel.2018.01.026)
Supplement: Document S2. Article plus Supplemental Information [file mmc12.pdf]

Article

Developmental Cell

Fat Body Cells Are Motile and Actively Migrate to Wounds to Drive Repair and Prevent Infection

Highlights

- Fat body cells actively migrate to wounds using a peristaltic mode of motility
- Fat body cells tightly seal the gap by forming lamellipodia around the wound margin
- Fat body cells collaborate with macrophages to clear wound debris
- Fat body cells locally release antimicrobial peptides at infected wounds

Authors

Anna Franz, Will Wood, Paul Martin

Correspondence

w.wood@ed.ac.uk (W.W.),
paul.martin@bristol.ac.uk (P.M.)

In Brief

Adipocytes and their fly equivalent, fat body cells, have been considered immotile, but Franz et al. now show the latter can actively migrate to wounds using a peristaltic-like “swimming” motility. Once there, they multitask to clear wound cell debris, plug the epithelial gap, and upregulate AMPs to prevent infection.

Franz et al., 2018, Developmental Cell *44*, 460–470
February 26, 2018 © 2018 The Author(s). Published by Elsevier Inc.
https://doi.org/10.1016/j.devcel.2018.01.026

CellPress

Developmental Cell
Article

Fat Body Cells Are Motile and Actively Migrate to Wounds to Drive Repair and Prevent Infection

Anna Franz,[1] Will Wood,[2,5,6,*] and Paul Martin[1,3,4,6,7,*]
[1]School of Biochemistry, Biomedical Sciences, University of Bristol, Bristol BS8 1TD, UK
[2]School of Cellular and Molecular Medicine, Biomedical Sciences, University of Bristol, Bristol BS8 1TD, UK
[3]School of Physiology, Pharmacology and Neuroscience, Biomedical Sciences, University of Bristol, Bristol BS8 1TD, UK
[4]School of Medicine, Cardiff University, Cardiff CF14 4XN, UK
[5]Present address: Medical Research Council Center for Inflammation Research, Queen's Medical Research Institute, University of Edinburgh, Edinburgh EH16 4TJ, UK
[6]Senior author
[7]Lead Contact
*Correspondence: w.wood@ed.ac.uk (W.W.), paul.martin@bristol.ac.uk (P.M.)
https://doi.org/10.1016/j.devcel.2018.01.026

SUMMARY

Adipocytes have many functions in various tissues beyond energy storage, including regulating metabolism, growth, and immunity. However, little is known about their role in wound healing. Here we use live imaging of fat body cells, the equivalent of vertebrate adipocytes in *Drosophila*, to investigate their potential behaviors and functions following skin wounding. We find that pupal fat body cells are not immotile, as previously presumed, but actively migrate to wounds using an unusual adhesion-independent, actomyosin-driven, peristaltic mode of motility. Once at the wound, fat body cells collaborate with hemocytes, *Drosophila* macrophages, to clear the wound of cell debris; they also tightly seal the epithelial wound gap and locally release antimicrobial peptides to fight wound infection. Thus, fat body cells are motile cells, enabling them to migrate to wounds to undertake several local functions needed to drive wound repair and prevent infections.

INTRODUCTION

There is a growing realization that adipocytes, once believed to act merely as local reservoirs of energy and to provide mechanical and thermal insulation, also have numerous other roles in various tissues in health and disease. These range from systemic metabolic and immune regulation through to key functions in tissue development and cancer progression (Hepler et al., 2017; Hoy et al., 2017; Maurizi et al., 2017; Rivera-Gonzalez et al., 2014). In the context of skin, there is a clear link between initial seeding of adipocyte precursors and subsequent dermal differentiation and hair follicle growth (Rivera-Gonzalez et al., 2014). However, rather little is known about the potential function of adipocytes in tissue repair. After skin wounding myofibroblasts have been shown to transdifferentiate into adipocytes (Plikus et al., 2017). Furthermore, adipocyte precursor cells are known to differentiate into mature adipocytes and these appear to contribute to repair because blocking their differentiation leads to defects in fibroblast migration and matrix deposition (Schmidt and Horsley, 2013). Other known functions of adipocytes include antimicrobial activities, since *Staphylococcus aureus* infection of otherwise healthy skin leads to rapid proliferation of dermal adipocytes, and impaired adipogenesis results in increased skin infections (Zhang et al., 2015).

The *Drosophila* fat body is considered to be equivalent to both the vertebrate adipocytes and liver, and is known to play many diverse systemic roles throughout all insect life stages. It regulates metabolism by actively sensing nutritional conditions and accordingly storing or releasing energy in the form of lipids, glycogen, and protein (Beller et al., 2010; Bi et al., 2012; Grönke et al., 2005, 2007). Importantly, fat storage in intracellular lipid droplets, and the mechanisms and key components responsible for stored-fat mobilization in the *Drosophila* fat body and mammalian adipocytes, appear to be evolutionarily conserved (Grönke et al., 2007). In addition to storing energy, the fat body also plays a central role in regulating systemic growth in response to nutrition. Upon sensing dietary amino acids, the fat body secretes several humoral factors, which control systemic growth of the animal (Britton and Edgar, 1998; Colombani et al., 2003; Delanoue et al., 2016; Géminard et al., 2009; Sousa-Nunes et al., 2011). This is achieved, in part, by the regulated secretion of insulin-like peptides by the insulin-producing cells of the brain (Géminard et al., 2009). Furthermore, the fat body is known also to play a crucial role in systemic immunity. Bacterial and fungal infections activate the Toll and IMD pathways in the fat body, resulting in the systemic expression and secretion of several antimicrobial peptides (AMPs), including Attacin (Buchon et al., 2014; Lemaitre and Hoffmann, 2007).

While major efforts have been made over the last few decades to elucidate the roles of the fat body in regulating metabolism, growth, and immunity, its potential role in wound repair has not been studied to date. Using live imaging of pupal epithelial wounds we show for the first time that pupal fat body cells (FBCs) are motile cells that actively migrate to wounds. We find that these giant cells move through the hemolymph toward the wound using an adhesion-independent, actomyosin-driven, peristaltic mode of motility. Once they have reached the wound,

This is an open access article under the CC BY license (http://creativecommons.org/licenses/by/4.0/).

FBCs assist hemocytes in clearing the wound of cell debris as well as sealing the epithelial wound gap and locally releasing AMPs to repair the wound and fight infection.

RESULTS

FBCs Are Motile and Migrate toward Wounds

To investigate the potential functions of FBCs during wound healing, we first studied their location and potential behaviors following tissue injury in pupae, since this developmental stage has proven ideal for live imaging of other wound healing events (Antunes et al., 2013; Weavers et al., 2016b). We found that 16-hr-old pupae contain large numbers of giant polyploid, dissociated FBCs that populate the body cavity (Figure 1A). To study the behaviors of FBCs following tissue injury by live imaging, we used a laser to induce small epithelial wounds in the ventral thorax of pupae, an area sparsely populated by FBCs (Movie S1). Nuclei were labeled with Histone-red fluorescent protein (RFP; bright red, condensed nuclei mark damaged epithelial cells in the wound area, while dimmer red nuclei mark the surrounding healthy epithelial cells; Figure S1) and FBCs were labeled with GFP (Figures 1B and 1C). Strikingly, we found that FBCs, previously thought to be immotile, were actually highly dynamic, and migrated rapidly toward wounds. Once at the wound site these cells remained tightly associated with the wound until closure, when they detached and actively migrated away (Figures 1B and 1C; Movie S2, first movie). When we compared small, medium, and large wounds (30–60, 60–90, and 90–120 μm in diameter, respectively; Figures 1D–1F; Movie S2, second, third, and fourth movies), we found that the frequency of FBC recruitment to wounds (Figure 1G), as well as the number of wound-associated FBCs (Figure 1H), positively correlated with the size of the wound: for small wounds, a single FBC generally plugged the wound, whereas in larger wounds up to 5 FBCs approached and associated with the wounded area (Figures 1D–1F, 1H, and Movie S2). The time of FBC arrival at the wound was variable, depending on their initial distance from the wound; some FBCs arrived after 10 min, with the average arrival time being around 1 hr after wounding, irrespective of wound size (Figure 1I). Once FBCs started contacting the wound area, they usually remained associated until reepithelialization was complete, resulting in a longer period of FBC-wound association in larger wounds with longer closure times (Figure 1I).

FBCs Migrate to Wounds with Directional Persistence

To test whether the recruitment of FBCs to wounds was driven by true directed migration and not just a random walk or passive fluid flow, we tracked individual cells in wounded and unwounded pupae (Figure 2A) and measured the directional persistence of the resulting tracks. Further analysis of these tracks showed an increase in the meandering index and a decrease in the angle of migration, together suggesting that wound-recruited FBCs responded to the wounds with high directional persistence (Figures 2B and 2C). The movement of FBCs to wounds is not due to passive flow of hemolymph toward the wound; this possibility has previously been ruled out by bead-tracking experiments following epithelial wounding in pupae where we saw no such flow (Weavers et al., 2016b). Moreover, we see no hemolymph leakage from wounds since laser wounding generally results in cuticular holes of <0.5 μm in diameter (Figure S1D). Next, we measured the speed of FBCs and found that they did not accelerate toward the wound; their meandering index was increased but their speed remained the same as in unwounded pupae until they reached the wound, when they decelerated and stopped (Figure 2D and Movie S2, first movie). Once the wound became fully occupied by one or more FBCs, late-arriving cells appeared unable to gain direct access because this space was occupied by earlier-arriving FBCs, but they often remained in the vicinity and circulated at the periphery (Movie S2, first movie; Figure S2A). Interestingly, both wound-recruited and late-arriving FBCs initially showed an increase in their meandering index (Figure S2B), suggesting that both cell populations respond equally to wound attractants.

FBC Migration Is Achieved via an Actomyosin-Driven "Peristaltic" Swimming Motion

Given our observation that *Drosophila* FBCs can actively migrate, we used live imaging of the actin cytoskeleton to understand the mechanism by which these cells power their migration. Most cells, whether in tissue culture or *in vivo* within tissues, migrate by adhering to, and crawling over, a substratum, often using actin-rich lamellipodia at their leading edges. By contrast, FBCs are not adherent to any epithelial surface; rather, they reside within the hemolymph (Figure 1A and Movie S1). To our surprise, live imaging of FBCs expressing GMA (GFP fused to the actin-binding domain of moesin) revealed that these cells were constantly undergoing actin-based contractile waves that initiated from the cortex of the cell center and extended to the rear of the cell, propelling them in the opposite direction (i.e., forward) in a peristaltic fashion. These waves occurred constantly within FBCs in unwounded pupae (Figure 3A; Movie S3, first movie) but upon wounding became highly directed with respect to the wound (Figures 3B and 3C; Movie S3, second movie). Using markers of the actin regulatory proteins, Fimbrin, Ena, and Fascin, we saw no sign of the more standard lamellipodial structures, observed for example in *Drosophila* macrophages (hemocytes), as they migrate to wounds (Stramer et al., 2005), as FBCs "swam" toward the wound (Movies S4 and S5). However, once they had reached the wound, FBCs started to form lamellipodia that extended around the wound margin (Movies S4 and S5).

In order to test whether motility of FBCs is indeed actomyosin driven, we expressed a dominant-negative version of Zipper (Myosin II heavy chain) tagged with YFP specifically in FBCs. During early pupal development, FBCs normally undergo an extensive remodeling, characterized by the dissociation of the fat body into single cells followed by their redistribution in the body cavity. This redistribution leads to the translocation of some cells into the anterior head capsule, which has previously been suggested to be driven by abdominal muscular contractions (Bond et al., 2011; Cherbas et al., 2003). Interestingly, we found that expression of dominant-negative Zipper-YFP only in FBCs led to a complete failure in FBC redistribution within the body cavity and translocation into the head (Figure 3D). Moreover, when we imaged and tracked these cells in the dorsal abdomen we found that their general motility was strongly reduced (Figures 3E and 3F; Movie S6). This suggests that the developmental process of FBC redistribution and translocation in pupae is not driven passively by muscular body contractions

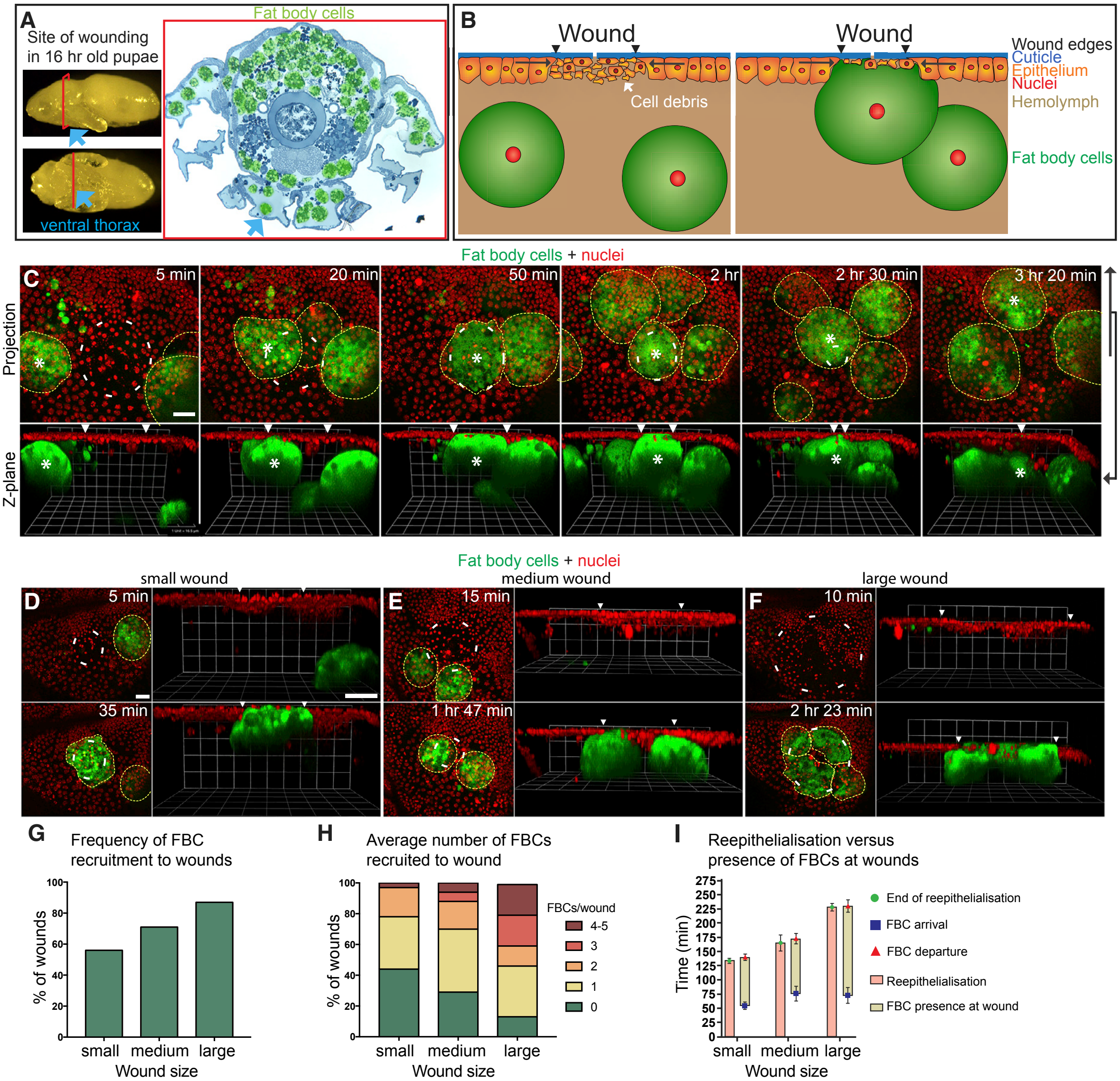

Figure 1. FBCs Actively Migrate toward Epithelial Wounds

(A) Images of pupae and methylene blue-stained section of the pupal thorax (FBCs false-colored green) showing FBC location and indicating site of laser wounding in the ventral thorax (blue arrows).

(B and C) Schematic (B) and time-lapse (C) images to illustrate FBC migration to a wound (projection, C top; Z plane, B and C bottom) in a c564-Gal4+UAS-GFP+Ubq>Histone-RFP pupa (epithelial nuclei in red; FBCs in green and outlined; asterisk labels wound-associated FBCs; arrowheads indicate wound margins). See also Movie S2, first movie, and Figure S1.

(D–H) Time lapse (D–F) and graphs (G and H) showing how FBCs are drawn to small, medium, and large wounds (30–60 μm, 60–90 μm, and 90–120 μm in diameter; n = 32, 12, and 15, respectively) in c564-Gal4+UAS-GFP+Ubq>Histone-RFP pupae (epithelial nuclei in red; FBCs in green and outlined). See also Movie S2, second, third, and fourth movies.

(I) Graph showing duration of reepithelialization (pink bar) and FBC presence (yellow bar) in small, medium, and large wounds (n = 17, 11, and 11, respectively; genotype as in D–H). Mean ± SEM.

Scale bars, 20 μm (C and D). (E) and (F) are the same magnification as (D).

but is instead an active process driven by actomyosin-dependent migration of FBCs. Similarly, expression of dominant-negative Zipper in FBCs completely blocked their ability to migrate to wounds in the ventral thorax (0% recruitment of FBCs to wounds, n = 72 wounds). Together, these data suggest that pupal FBCs are indeed motile cells, which migrate using an adhesion-independent, actomysin-driven peristaltic mode of motility during both their developmental dispersal and their recruitment to wounds.

Macrophages and FBCs Together Displace and Phagocytose Cellular Debris from the Wound Site

Previous studies have shown that hemocytes, the equivalent of macrophages in *Drosophila*, are actively drawn to wound sites

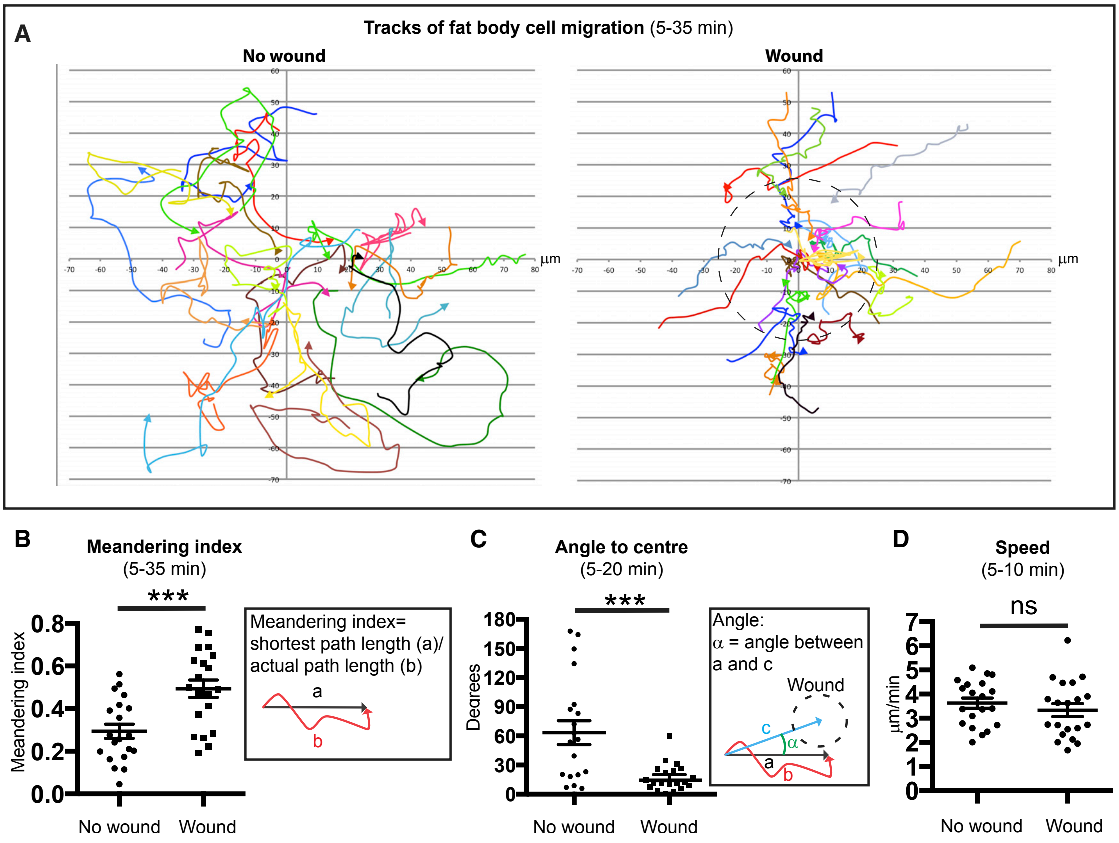

Figure 2. FBCs Migrate to Wounds with Directional Persistence

(A–D) Migration tracks of FBCs (A) and quantification of meandering index (B), angle (C), and speed (D) of FBC migration in c564-Gal4+UAS-GFP+Ubq>Histone-RFP unwounded or wounded pupae (n = 20 and 20); only analyzing cells that passed through a circular area of 25-μm radius from center within 30-min time window. See also Figure S2. Mean ± SEM. ns, $p > 0.05$; ***$p < 0.001$ (Student's t test).

in embryos and pupae (Stramer et al., 2005; Weavers et al., 2016b), much as innate immune cells are drawn to wounds in vertebrates (Eming et al., 2017). Interestingly, larval hemocytes have been shown to collaborate with and even communicate with FBCs through cytokine release in response to bacterial infections, leading to a scenario whereby hemocytes phagocytose bacteria while FBCs produce AMPs systemically, but these AMP levels are significantly reduced in the absence of hemocytes (Shia et al., 2009). To investigate whether hemocytes and FBCs interact with one another during wound healing, we wounded pupae in which both hemocytes and FBCs were labeled with cytosolic GFP and nuclear RFP. Both cell types migrated at approximately the same speed, 2.5–3.5 μm/min (Movie S7; Figures 4A and 2D; Stramer et al., 2005), although in general, due to their proximity to the wound and increased numbers, hemocytes often arrived before FBCs (Figure 4A). We see the same if these two lineages are labeled with complementary cytosolic GFP and mCherry tags (Figure S3 and Movie S8). Interestingly, most hemocytes were swept aside as the first FBC approached the wound (Movies S7 and S8). To test whether FBC recruitment might be dependent on the presence of hemocytes at the wound, we genetically ablated hemocytes through lineage-specific expression of apoptosis-inducing Reaper for 16 hr before wounding. This loss of hemocytes did not significantly alter the frequency of FBC recruitment to wounds (70% and 60% of wounds with or without hemocyte ablation, n = 11 and 9, respectively; Figures 4B and 4C; Movie S9), suggesting that FBCs are not drawn to wounds by attractant signals released by hemocytes.

Given our finding that FBCs are motile and rapidly migrate to wounds, next we wanted to investigate what local functions they might play during wound healing. Efficient wound repair requires the clearance of wound debris from the wound site, which is known to be, in part, orchestrated by hemocytes through phagocytosis (Weavers et al., 2016a). Interestingly, we noticed that, when we ablated hemocytes, the majority of cellular debris at the wound (visualized as bright Histone-RFP-labeled nuclei of damaged epithelial cells) was swept aside by the incoming FBCs (Figures 4B and 4C; Movie S9). In the presence of hemocytes, this clearance of cell debris away from the wound site by FBCs also occurred, albeit to a lesser extent, and was accompanied by engulfment of the debris by hemocytes (Movie S2). We also observed phagocytic cup formation and subsequent engulfment of debris at the wound site by FBCs in 35% of small and 75% of large wounds, which contained wound-recruited FBCs (n = 17 and 12, respectively, Figure 4D). Thus FBCs, in concert with hemocytes, appear to play an important local function in clearing cell debris during wound repair: FBCs physically clear the wound

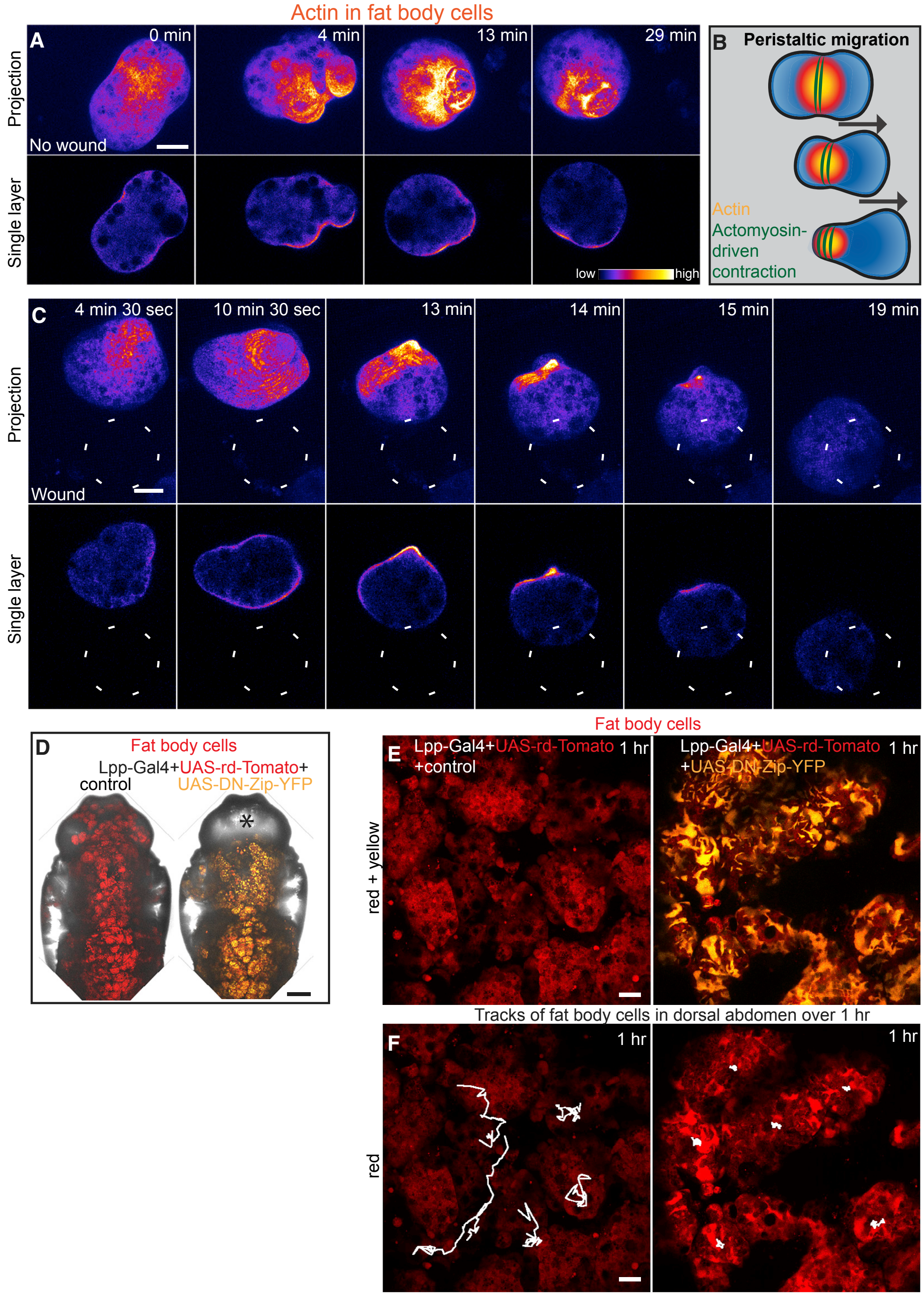

Figure 3. FBCs Actively Migrate toward Wounds Using a Novel Actomyosin-Driven Peristaltic Mode of Motility

(A–C) Time lapse (projection at top, single plane beneath) showing actin dynamics in FBCs within unwounded (A) or wounded (C) Lpp-Gal4+UAS-GMA pupae (GMA shown in ImageJ LUT Fire). Location of the wound indicated by white dashed circle. Schematic illustrating peristaltic migration (B). See also Movie S3.

(D–F) Low-magnification (D) and high-magnification (E and F) images of Lpp-Gal4+UAS-rd-Tomato+control or +UAS-DN-Zip-YFP pupae; FBCs in red, DN-Zip-YFP in yellow; asterisk marks absence of FBCs in head (D); 1-hr migration tracks of FBCs, white lines (F). See also Movie S6 and Figure S4. Control = w67. Scale bars, 20 μm (A, C, E, and F) and 200 μm (D).

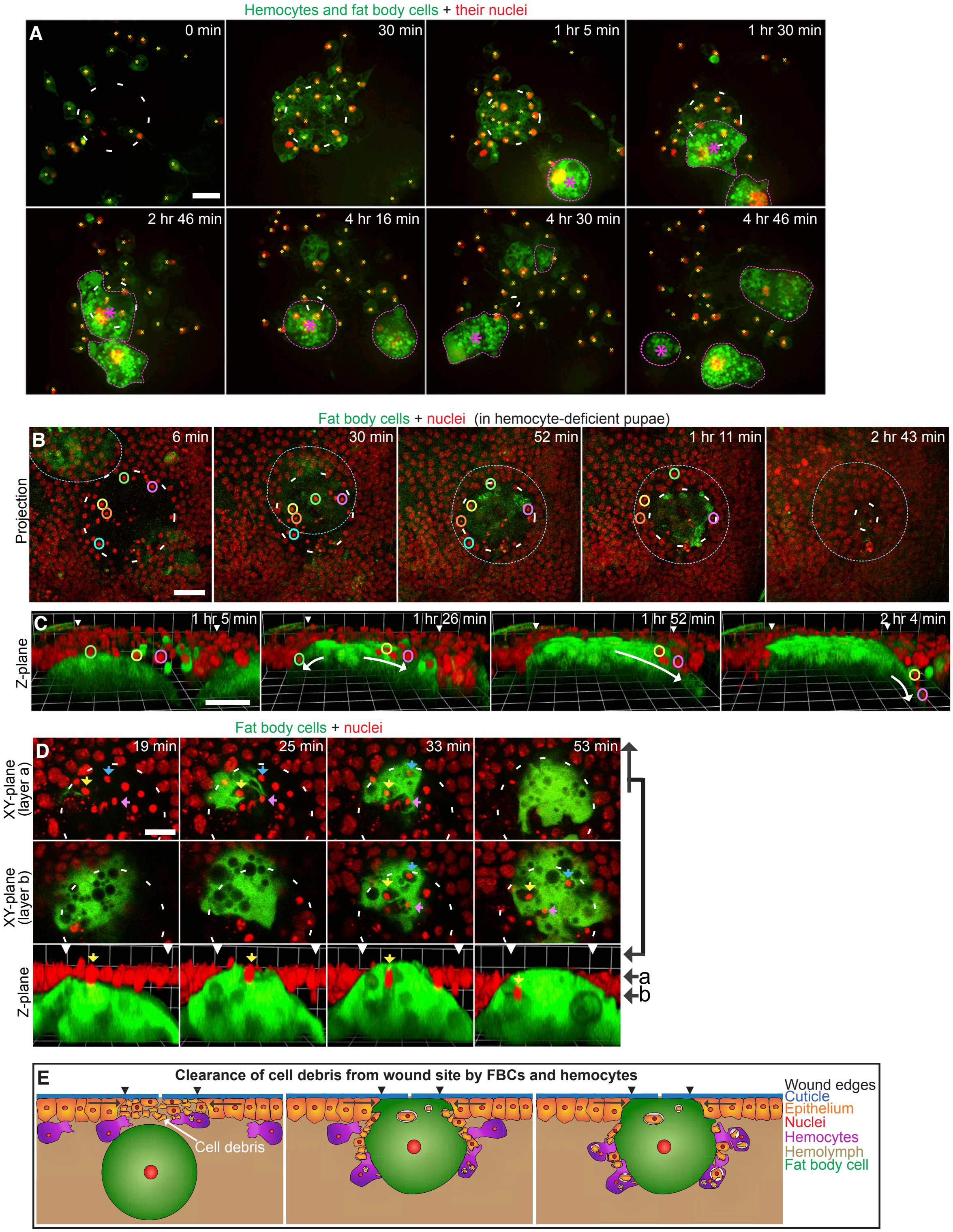

Figure 4. FBCs and Hemocytes Together Clear the Wound of Debris

(A) Time lapse of hemocyte and FBC recruitment to a wound in a srp-Gal4+c564-Gal4+UAS-GFP+UAS-Red-Stinger pupa (hemocytes are small green cells with red nuclei and yellow asterisks; FBCs are large green cells with red nuclei and outlined; purple asterisk labels wound-associated FBCs). See also Movie S7 and Figure S3.

(B and C) Time-lapse sequences of wounded srp-GMA+Ubq>Histone-RFP pupae (FBC in green and outlined; epithelial nuclei in red; colored circles highlight some nuclei of necrotic epithelial cells) expressing srp-Gal4+UAS-Reaper+tubGal80ts for 16 hr before wounding at the restrictive temperature to ablate hemocytes. See also Movie S9.

(legend continued on next page)

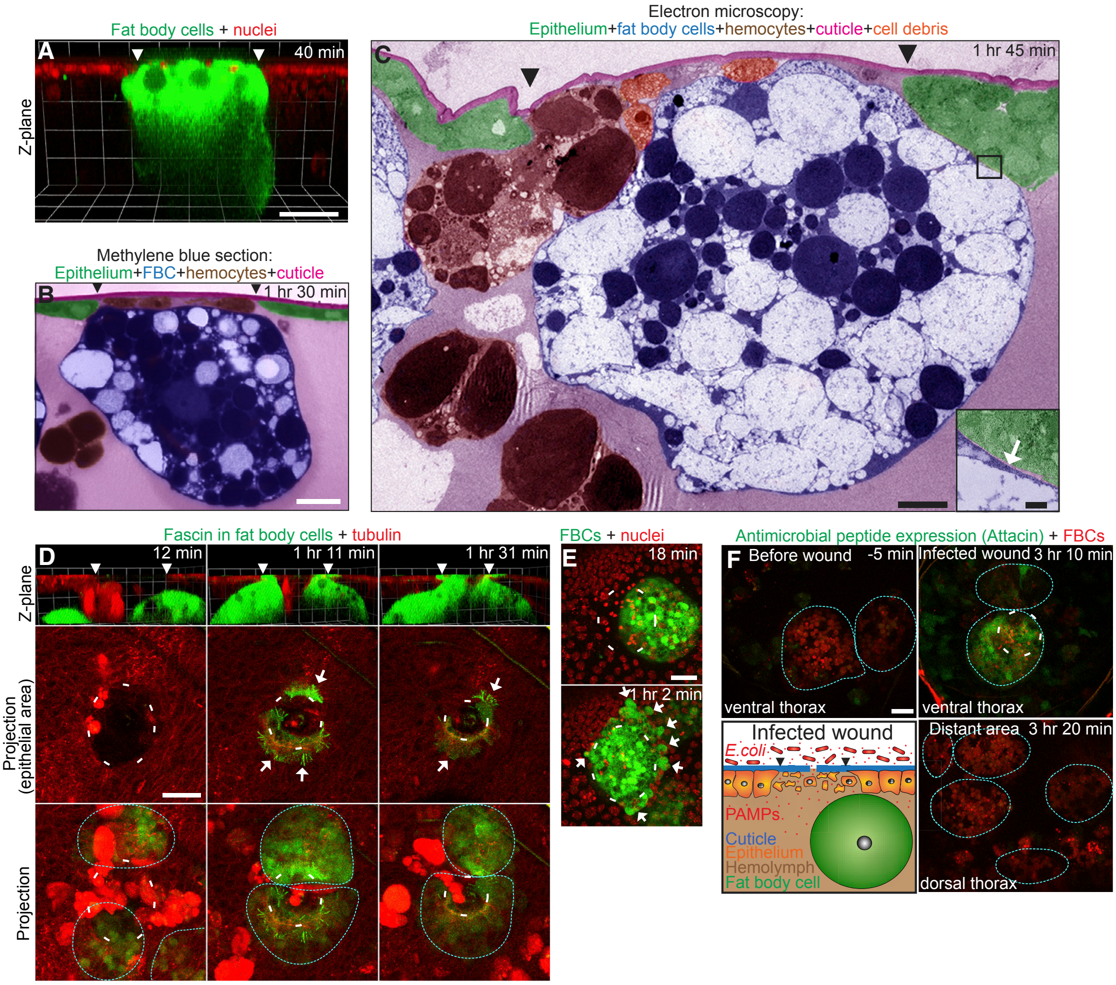

Figure 5. FBCs Seal the Wound and Locally Produce AMPs

(A) Time lapse (single frame) of wound-plugging by an FBC in a c564-Gal4+UAS-GFP+Ubq>Histone-RFP pupa (FBC in green; epithelial nuclei in red).

(B and C) Methylene blue-stained resin section (B) and transmission electron microscopy (C) images of FBC plugging the wound (different cell types are shown false-colored, as indicated).

(D) Time lapse of lamellipodia formation by FBCs at the wound of a c564-Gal4+UAS-GFP-Fascin+Ubq>RFP-tubulin pupa (epithelium in red; Fascin in green, Fascin-rich protrusions indicated with arrows). See also Movie S5.

(E) Time lapse of FBC blebbing at a wound in a c564-Gal4+UAS-GFP+Ubq>Histone-RFP pupa (epithelial nuclei in red; FBCs in green; blebs indicated with arrows). See also Movie S2, second movie.

(F) Time lapse of local Attacin expression in FBCs in an Attacin>GFP+Lpp-Gal4+UAS-myr-td-Tom pupa after wounding and exposure to RFP-*E. coli* for 5 min (FBCs in red and outlined; Attacin expression, green). Schematic illustrating experimental setup. See also Movie S10.

Scale bars, 20 μm (A, B, and D–F), 5 μm (C) and 500 nm (C, insert).

site of cell debris by displacing it to the wound periphery, where hemocytes, and to a lesser extent FBCs, take up the debris by phagocytosis (Figure 4E).

FBCs Multitask at the Wound Site to Both Seal the Gap and Produce AMPs to Stave Off Infection

Next, we wanted to investigate whether, in addition to wound repair, FBCs might play local functions in fighting wound infection. Given the large size of FBCs and their apparent tight association with the wound throughout closure, we wondered whether they might play a role in plugging the wound to prevent entrance of pathogens and leakage of tissue fluids, much as a clot in a vertebrate wound. Light and transmission electron microscopy revealed an extremely tight seal between wound-associated FBCs and the epithelial wound margin (Figures 5A–5C; Movie S2), leaving a gap of less than 20 nm (see inset Figure 5C),

(D) Time lapse (two X/Y planes at top and middle and Z plane at bottom) showing phagocytic uptake of debris by an FBC in a wounded c564-Gal4+UAS-GFP+Ubq>Histone-RFP pupa (FBCs in green; epithelial nuclei in red; colored arrows highlight some nuclei of necrotic epithelial cells to aid tracking).

(E) Schematic illustrating collaborative clearance of cell debris from wound site by FBCs and hemocytes.

Scale bars, 20 μm (A–C) and 10 μm (D).

which would be too small for bacteria to pass through. In order to understand how these cells are able to achieve such a tight association, we again turned to live imaging of the actin dynamics within these cells as they arrive at the wound site. Expressing fluorescently tagged forms of the actin regulatory proteins Fascin, Fimbrin, and Ena revealed that, although FBCs moved to the wound without the use of lamellipodia or blebbing, once at the wound site, all co-operating cells extended lamellipodial protrusions from their apical surfaces that reached around and out of the wound margin, to form a tight seal (Figure 5D; Movies S4 and S5). These dynamic lamellipodia remained in a ring formation around the closing wound edge, sealing off the wound from the body cavity of the animal until reepithelialization was complete. Throughout this period, FBCs within the vicinity of the wound produced extensive blebs as if jostling to become more firmly wedged into the wound gap and form an effective plug (Figure 5E; Movie S2, second movie). Interestingly, this plugging of the wound may come with some cost; since expressing dominant-negative Zipper specifically in FBCs to prevent their recruitment to wounds (see earlier) resulted in significantly faster wound reepithelialization (Figure S4). This suggests that the presence of FBCs at the wound site may partially physically obstruct epithelial closure.

Drosophila FBCs have been shown to systemically produce a variety of AMPs following infection with a variety of pathogens (Buchon et al., 2014; Lemaitre and Hoffmann, 2007). We wondered whether, since wounding is generally associated with infection, local delivery of AMPs to the wound might be another function of FBCs recruited to wounds. To test this, we wounded pupae expressing a reporter of one of these AMPs, Attacin, and then briefly added RFP-labeled *Escherichia coli* to the wound prior to live imaging. Our laser wounding usually only results in a very small hole in the cuticle of <0.5 μm in diameter (Figure S1D) and so bacteria generally fail to enter the wound but their pathogen-associated molecular patterns (PAMPs) can (schematic in Figure 5F and data not shown). Wounding in the absence of an infection did not induce an upregulation of Attacin in FBCs. In contrast with this, within 60–90 min of making an infected wound, we saw an upregulation of Attacin in a few epithelial cells near the wound, as well as in the FBCs that plugged the wound and those in close proximity, up to 50 μm away (Figure 5F and Movie S10). This was clearly a local response by the FBCs since it did not occur in FBCs distant from the wound (Figure 5F). This shows that, following wound infection, FBCs are able to detect bacteria at the epithelial breach and locally deliver AMPs. Thus, FBCs, together with hemocytes, appear to play important local functions in combating wound infection: FBCs plug the wound gap, thereby forming a barrier that might prevent entry of bacteria and locally deliver AMPs to fight off pathogens, while hemocytes clear bacteria by phagocytosis.

DISCUSSION

Our data show that FBCs, *Drosophila* adipocytes, are recruited to wounds in pupae where they have multiple local roles in wound healing. The observation that FBCs are motile cells that actively migrate to wounds is unexpected and has not previously been made for adipocytes in any other organism. However, our findings raise the interesting question as to whether vertebrate adipocytes might also have the capacity to migrate. In that regard, a recent mammalian wound study found that adipocytes repopulate murine wounds, and suggested that some may have migrated from distant sites (Schmidt and Horsley, 2013). It will be fascinating to discover whether some sub-populations of vertebrate adipocytes are indeed motile and whether they utilize similar migratory strategies to those highlighted in *Drosophila* FBCs.

The mode of motility we observe for FBCs moving through the hemolymph to wounds is unusual, since it does not appear to involve the use of standard lamellipodia or blebs, utilized by most known migrating cells as they crawl in an adhesion-dependent fashion over substrates and through a milieu of extracellular matrix. Adhesion-independent migration has recently emerged as an alternative migration mode that has now been described for several other types of cells, including ameba, lymphocytes, and some cancer cells (Paluch et al., 2016). Four models have been proposed for adhesion-independent migration: force transmission driven by "chimneying" between two opposing substrate faces, the intercalation of lateral cell protrusions with gaps in the substrate, non-specific friction between cell and substrate, and swimming by noncyclic cell shape deformations (Paluch et al., 2016). Only the last of these is entirely independent of any interactions with (or close proximity to) a solid substrate and hence best describes our observation of the migration of FBCs through hemolymph to wounds, since we do not see significant interactions of these cells with any substrate or other cells as they migrate. Similar to FBCs, several other cell types have been reported to migrate by swimming, when they are required to move through viscous fluid: amebae and neutrophils have been shown to swim when in viscous solution (Barry and Bretscher, 2010) and lymphocytes are known to migrate using contraction waves when in suspension (Haston and Shields, 1984). However, the exact mechanism by which these swimming cells generate internal forces and how these forces are transduced to the extracellular environment to generate forward movement is still unknown. A recent study has shed some light on how internal forces are generated during another type of adhesion-independent migration; it showed that the migration of Walker carcinoma cells in confinement is driven by cyclical rearward flow of cortical actin that is coupled to the substrate through friction. This migration depends on the contractility of cortical actin at the rear of the cells (Bergert et al., 2015). Moreover, rearward flow of cortical actin has also been described for the oscillatory behavior of detached cells and cell fragments (Paluch et al., 2005), as well as for the stable-bleb cell migration of zebrafish germ layer progenitor cells (Ruprecht et al., 2015). This is strikingly similar to the rearward peristaltic actin waves we observe in FBCs migrating to wounds, suggesting that this could be the mechanism of force generation in FBCs also.

However, it still remains unclear how such an intracellular force might be transduced to the extracellular environment to drive forward movement of FBCs. It has previously been presumed that, while swimming works for large multicellular organisms, it cannot operate at the microscopic cell level, where viscous forces are many orders of magnitude higher than inertial forces (i.e., at low Reynold's number; Purcell, 1977) and hence geometrically reciprocal cell shape changes may not generate propulsive forces (Paluch et al., 2016). However, this view has been

challenged and may only be true for simple Newtonian fluids, like water (Qiu et al., 2014), which the hemolymph that FBCs swim through is clearly not. Moreover, swimming in a non-Newtonian fluid is thought to be possible if the cell shape changes of migrating cells are nonreciprocal, which might be true for FBCs migrating to wounds. It is also possible that FBCs, in addition to swimming, make use of other mechanisms to migrate. The hemolymph is relatively densely packed with cells including hemocytes and other FBCs (see Figure 1A), and FBCs are adjacent to the epithelium and muscle, depending on the location in the body. Although we have not observed contacts, it is possible that the close proximity of FBCs with other cells and tissues en route to a wound might enable them to occasionally generate additional frictional forces like the ones reported for non-adherent Walker cells migrating in a confined microfluidics channel (Bergert et al., 2015), which may also contribute to their swimming motility.

Our study shows that FBCs play multiple local roles in driving wound repair and preventing wound infection. We wonder whether some of these local functions might also partially extrapolate to the vertebrate wound scenario. *Drosophila* FBCs have long been known to systemically produce a variety of AMPs following infection (Buchon et al., 2014; Lemaitre and Hoffmann, 2007) and our study reveals that, during wound infection, FBCs migrate to wounds to release AMPs locally. A recent study has shown that mouse adipocytes are able to produce AMPs following bacterial skin infections (Zhang et al., 2015). Hence, it would be interesting to examine whether mammalian adipocytes, like *Drosophila* FBCs, play a local role during wound healing in delivering AMPs to fight wound infection.

Given our finding that hemocytes and FBCs collaborate during the wound repair process to clear cell debris and fight infection, it is tempting to speculate that these two cell types communicate with each other during vertebrate wound healing also. Interestingly, in recent years several mammalian studies have uncovered complex interactions between adipocytes and macrophages in white adipose tissue (WAT), with important implications for tissue regeneration and disease (Shook et al., 2016). One example is obesity-induced inflammation and insulin resistance, where, upon overnutrition, the adipocytes in visceral WAT are thought to release chemokines to stimulate macrophage recruitment into fat tissue, leading to smoldering inflammation and subsequently insulin resistance (Osborn and Olefsky, 2012). This is believed to be due to proinflammatory macrophages releasing cytokines that attenuate insulin signaling in various cell types, including adipocytes (Osborn and Olefsky, 2012). In support of these mammalian reports, a recent study in the fly showed that animals fed a lipid-rich diet display reduced insulin sensitivity and lifespan, and both of these effects are mediated by hemocytes (Woodcock et al., 2015).

Thus interactions between adipocytes and immune cells appear to be key in many diseases, including type 2 diabetes, and we believe that important insights into these links may be provided by future studies of the functional relationship and communication between FBCs and hemocytes during pupal wound repair in flies.

Our studies in *Drosophila* pupae flag up novel behaviors and functions for FBCs in *Drosophila* and open up genetic opportunities to further our understanding of the important roles played by adipocytes in repair and regeneration.

STAR★METHODS

Detailed methods are provided in the online version of this paper and include the following:

- KEY RESOURCES TABLE
- CONTACT FOR REAGENT AND RESOURCE SHARING
- EXPERIMENTAL MODEL AND SUBJECT DETAILS
  - Fly Stocks and Preparation
- METHODS DETAILS
  - Microscopy and Wounding
  - Image Processing and Analysis
- QUANTIFICATION AND STATISTICAL ANALYSIS

SUPPLEMENTAL INFORMATION

Supplemental Information includes four figures, one table, and ten movies and can be found with this article online at https://doi.org/10.1016/j.devcel.2018.01.026.

ACKNOWLEDGMENTS

We thank Deborah Carter for technical support with electron microscopy; Naren Srinivasan, Maaike van den Berg, David Gurevich, Helen Weavers, Brian Stramer, and Jordan Raff for comments on the manuscript; Ilona Aylott for her help with Illustrator; and members of W.W.'s and P.M.'s labs for helpful discussion. We also thank Stephen Cross (funded by EBI, Wellcome Trust ISSF Award) and the rest of the team of the Wolfson Bioimaging Facility (Bristol, UK) for their help with imaging and image analysis, as well as Pierre Leopold (Nice University, France), Nicolas Buchon (Cornell University, USA), Alex Gould (The Francis Crick Institute, UK), Katja Bruckner (UCSF, USA), Brian Stramer (Kings College, UK), Jordan Raff (Oxford University, UK), Caetano Reis e Sousa (The Francis Crick Institute, UK), the Vienna *Drosophila* Resource Center (Vienna, Austria), and the Bloomington *Drosophila* Stock Center (Indiana, USA) for fly stocks. This work was funded by an MRC Program Grant to P.M. and W.W. (MR/J002577/1) and Wellcome Trust Investigator and Fellowship Awards to P.M. and W.W.

AUTHOR CONTRIBUTIONS

A.F. designed and performed the experiments, and A.F., W.W., and P.M. designed the study and wrote the manuscript.

DECLARATION OF INTERESTS

The authors declare no competing interests.

Received: June 29, 2017
Revised: December 4, 2017
Accepted: January 29, 2018
Published: February 26, 2018

REFERENCES

Antunes, M., Pereira, T., Cordeiro, J.V., Almeida, L., and Jacinto, A. (2013). Coordinated waves of actomyosin flow and apical cell constriction immediately after wounding. J. Cell Biol. *202*, 365–379.

Barolo, S., Castro, B., and Posakony, J.W. (2004). New *Drosophila* transgenic reporters: insulated P-element vectors expressing fast-maturing RFP. Biotechniques *36*, 436–440, 442.

Barry, N.P., and Bretscher, M.S. (2010). *Dictyostelium* amoebae and neutrophils can swim. Proc. Natl. Acad. Sci. USA *107*, 11376–11380.

Basto, R., Brunk, K., Vinadogrova, T., Peel, N., Franz, A., Khodjakov, A., and Raff, J.W. (2008). Centrosome amplification can initiate tumorigenesis in flies. Cell *133*, 1032–1042.

Beller, M., Bulankina, A.V., Hsiao, H.-H.H., Urlaub, H., Jäckle, H., and Kühnlein, R.P. (2010). PERILIPIN-dependent control of lipid droplet structure and fat storage in *Drosophila*. Cell Metab. *12*, 521–532.

Bergert, M., Erzberger, A., Desai, R.A., Aspalter, I.M., Oates, A.C., Charras, G., Salbreux, G., and Paluch, E.K. (2015). Force transmission during adhesion-independent migration. Nat. Cell Biol. *17*, 524–529.

Bi, J., Xiang, Y., Chen, H., Liu, Z., Grönke, S., Kühnlein, R.P., and Huang, X. (2012). Opposite and redundant roles of the two *Drosophila* perilipins in lipid mobilization. J. Cell Sci. *125*, 3568–3577.

Bond, N.D., Nelliot, A., Bernardo, M.K., Ayerh, M.A., Gorski, K.A., Hoshizaki, D.K., and Woodard, C.T. (2011). βFTZ-F1 and matrix metalloproteinase 2 are required for fat-body remodeling in *Drosophila*. Dev. Biol. *360*, 286–296.

Britton, J.S., and Edgar, B.A. (1998). Environmental control of the cell cycle in *Drosophila*: nutrition activates mitotic and endoreplicative cells by distinct mechanisms. Development *125*, 2149–2158.

Brückner, K., Kockel, L., Duchek, P., Luque, C.M., Rørth, P., and Perrimon, N. (2004). The PDGF/VEGF receptor controls blood cell survival in *Drosophila*. Dev. Cell *7*, 73–84.

Buchon, N., Silverman, N., and Cherry, S. (2014). Immunity in *Drosophila* melanogaster–from microbial recognition to whole-organism physiology. Nat. Rev. Immunol. *14*, 796–810.

Cherbas, L., Hu, X., Zhimulev, I., Belyaeva, E., and Cherbas, P. (2003). EcR isoforms in *Drosophila*: testing tissue-specific requirements by targeted blockade and rescue. Development *130*, 271–284.

Colombani, J., Raisin, S., Pantalacci, S., Radimerski, T., Montagne, J., and Léopold, P. (2003). A nutrient sensor mechanism controls *Drosophila* growth. Cell *114*, 739–749.

Dawes-Hoang, R.E., Parmar, K.M., Christiansen, A.E., Phelps, C.B., Brand, A.H., and Wieschaus, E.F. (2005). *folded gastrulation*, cell shape change and the control of myosin localization. Development *132*, 4165–4178.

Delanoue, R., Meschi, E., Agrawal, N., Mauri, A., Tsatskis, Y., McNeill, H., and Leopold, P. (2016). *Drosophila* insulin release is triggered by adipose Stunted ligand to brain Methuselah receptor. Science *353*, 1553–1556.

Dutta, D., Bloor, J.W., Ruiz-Gomez, M., VijayRaghavan, K., and Kiehart, D.P. (2002). Real-time imaging of morphogenetic movements in *Drosophila* using Gal4-UAS-driven expression of GFP fused to the actin-binding domain of moesin. Genesis *34*, 146–151.

Eming, S.A., Wynn, T.A., and Martin, P. (2017). Inflammation and metabolism in tissue repair and regeneration. Science *356*, 1026–1030.

Gates, J., Mahaffey, J.P., Rogers, S.L., Emerson, M., Rogers, E.M., Sottile, S.L., Van Vactor, D., Gertler, F.B., and Peifer, M. (2007). Enabled plays key roles in embryonic epithelial morphogenesis in *Drosophila*. Development *134*, 2027–2039.

Géminard, C., Rulifson, E.J., and Léopold, P. (2009). Remote control of insulin secretion by fat cells in *Drosophila*. Cell Metab. *10*, 199–207.

Grönke, S., Mildner, A., Fellert, S., Tennagels, N., Petry, S., Müller, G., Jäckle, H., and Kühnlein, R.P. (2005). Brummer lipase is an evolutionary conserved fat storage regulator in *Drosophila*. Cell Metab. *1*, 323–330.

Grönke, S., Müller, G., Hirsch, J., Fellert, S., Andreou, A., Haase, T., Jäckle, H., and Kühnlein, R.P. (2007). Dual lipolytic control of body fat storage and mobilization in *Drosophila*. PLoS Biol. *5*, e137.

Gyoergy, A., Roblek, M., Ratheesh, A., Valoskova, K., Belyaeva, V., Wachner, S., Matsubayashi, Y., Sanchez-Sanchez, B.J., Stramer, B., and Siekhaus, D.E. (2018). Tools allowing independent visualization and genetic manipulation of *Drosophila melanogaster* macrophages and surrounding tissues. G3 (Bethesda). https://doi.org/10.1534/g3.117.300452.

Haston, W.S., and Shields, J.M. (1984). Contraction waves in lymphocyte locomotion. J. Cell Sci. *68*, 227–241.

Hepler, C., Vishvanath, L., and Gupta, R.K. (2017). Sorting out adipocyte precursors and their role in physiology and disease. Genes Dev. *31*, 127–140.

Hoy, A.J., Balaban, S., and Saunders, D.N. (2017). Adipocyte-tumor cell metabolic crosstalk in breast cancer. Trends Mol. Med. *23*, 381–392.

Kambris, Z., Brun, S., Jang, I.H., Nam, H.J., Romeo, Y., Takahashi, K., Lee, W.J., Ueda, R., and Lemaitre, B. (2006). *Drosophila* immunity: a large-scale in vivo RNAi screen identifies five serine proteases required for Toll activation. Curr. Biol. *16*, 808–813.

Lemaitre, B., and Hoffmann, J. (2007). The host defense of *Drosophila melanogaster*. Annu. Rev. Immunol. *25*, 697–743.

Maurizi, G., Della Guardia, L., Maurizi, A., and Poloni, A. (2017). Adipocytes properties and crosstalk with immune system in obesity-related inflammation. J. Cell Physiol. *233*, 88–97.

Moreira, S., Stramer, B., Evans, I., Wood, W., and Martin, P. (2010). Prioritization of competing damage and developmental signals by migrating macrophages in the *Drosophila* embryo. Curr. Biol. *20*, 464–470.

Osborn, O., and Olefsky, J.M. (2012). The cellular and signaling networks linking the immune system and metabolism in disease. Nat. Med. *18*, 363–374.

Paluch, E., Piel, M., Prost, J., Bornens, M., and Sykes, C. (2005). Cortical actomyosin breakage triggers shape oscillations in cells and cell fragments. Biophys. J. *89*, 724–733.

Paluch, E.K., Aspalter, I.M., and Sixt, M. (2016). Focal adhesion-independent cell migration. Annu. Rev. Cell Dev. Biol. *32*, 469–490.

Plikus, M.V., Guerrero-Juarez, C.F., Ito, M., Li, Y., Dedhia, P.H., Zheng, Y., Shao, M., Gay, D.L., Ramos, R., His, T.-C., et al. (2017). Regeneration of fat cells from myofibroblasts during wound healing. Science *355*, 748–752.

Purcell, E.M. (1977). Life at low Reynold's number. Am. J. Phys. *45*, 3–11.

Qiu, T., Lee, T.C., Mark, A.G., Morozov, K.I., Munster, R., Mierka, O., Turek, S., Leshansky, A.M., and Fischer, P. (2014). Swimming by reciprocal motion at low Reynolds number. Nat. Commun. *5*, 5119.

Razzell, W., Evans, I.R., Martin, P., and Wood, W. (2013). Calcium flashes orchestrate the wound inflammatory response through DUOX activation and hydrogen peroxide release. Curr. Biol. *23*, 424–429.

Rivera-Gonzalez, G., Shook, B., and Horsley, V. (2014). Adipocytes in skin health and disease. Cold Spring Harb. Perspect. Med. *4*.

Royou, A., Field, C., Sisson, J.C., Sullivan, W., and Karess, R. (2004). Reassessing the role and dynamics of nonmuscle myosin II during furrow formation in early *Drosophila* embryos. Mol. Biol. Cell *15*, 838–850.

Ruprecht, V., Wieser, S., Callan-Jones, A., Smutny, M., Morita, H., Sako, K., Barone, V., Ritsch-Marte, M., Sixt, M., Voituriez, R., et al. (2015). Cortical contractility triggers a stochastic switch to fast amoeboid cell motility. Cell *160*, 673–685.

Schmidt, B.A., and Horsley, V. (2013). Intradermal adipocytes mediate fibroblast recruitment during skin wound healing. Development *140*, 1517–1527.

Shia, A.K., Glittenberg, M., Thompson, G., Weber, A.N., Reichhart, J.M., and Ligoxygakis, P. (2009). Toll-dependent antimicrobial responses in *Drosophila* larval fat body require Spatzle secreted by haemocytes. J. Cell Sci. *122*, 4505–4515.

Shook, B., Rivera Gonzalez, G., Ebmeier, S., Grisotti, G., Zwick, R., and Horsley, V. (2016). The role of adipocytes in tissue regeneration and stem cell niches. Annu. Rev. Cell Dev. Biol. *32*, 609–631.

Sousa-Nunes, R., Yee, L., and Gould, A.P. (2011). Fat cells reactivate quiescent neuroblasts via TOR and glial insulin relays in *Drosophila*. Nature *471*, 508–512.

Stramer, B., Wood, W., Galko, M.J., Redd, M.J., Jacinto, A., Parkhurst, S.M., and Martin, P. (2005). Live imaging of wound inflammation in *Drosophila* embryos reveals key roles for small GTPases during in vivo cell migration. J. Cell Biol. *168*, 567–573.

Tzou, P., Ohresser, S., Ferrandon, D., Capovilla, M., Reichhart, J.M., Lemaitre, B., Hoffmann, J.A., and Imler, J.L. (2000). Tissue-specific inducible expression of antimicrobial peptide genes in *Drosophila* surface epithelia. Immunity *13*, 737–748.

Vlisidou, I., Dowling, A.J., Evans, I.R., Waterfield, N., ffrench-Constant, R.H., and Wood, W. (2009). *Drosophila* embryos as model systems for monitoring bacterial infection in real time. PLoS Pathog. *5*, e1000518.

Weavers, H., Evans, I.R., Martin, P., and Wood, W. (2016a). Corpse engulfment generates a molecular memory that primes the macrophage inflammatory response. Cell *165*, 1658–1671.

Weavers, H., Liepe, J., Sim, A., Wood, W., Martin, P., and Stumpf, M.P. (2016b). Systems analysis of the dynamic inflammatory response to tissue damage reveals spatiotemporal properties of the wound attractant gradient. Curr. Biol. *26*, 1975–1989.

Woodcock, K.J., Kierdorf, K., Pouchelon, C.A., Vivancos, V., Dionne, M.S., and Geissmann, F. (2015). Macrophage-derived upd3 cytokine causes impaired glucose homeostasis and reduced lifespan in *Drosophila* fed a lipid-rich diet. Immunity *42*, 133–144.

Zanet, J., Stramer, B., Millard, T., Martin, P., Payre, F., and Plaza, S. (2009). Fascin is required for blood cell migration during *Drosophila* embryogenesis. Development *136*, 2557–2565.

Zhang, L.J., Guerrero-Juarez, C.F., Hata, T., Bapat, S.P., Ramos, R., Plikus, M.V., and Gallo, R.L. (2015). Innate immunity. Dermal adipocytes protect against invasive *Staphylococcus aureus* skin infection. Science *347*, 67–71.

STAR★METHODS

KEY RESOURCES TABLE

REAGENT or RESOURCE	SOURCE	IDENTIFIER
Bacterial and Virus Strains		
E.coli: RFP-*E.coli*	Vlisidou et al., 2009	N/A
Experimental Models: Organisms/Strains		
D. melanogaster: w67	Gift from Jordan Raff	N/A
D. melanogaster: Lpp-Gal4	Gift from Pierre Leopold	N/A
D. melanogaster: c564-Gal4	Kambris et al., 2006	N/A
D. melanogaster: srp-Gal4	Brückner et al., 2004	N/A
D. melanogaster: tub-Gal80ts	Bloomington Drosophila stock center	RRID: BDSC7017
D. melanogaster: UAS-GFP-Ena	Gates et al., 2007	N/A
D. melanogaster: UAS-Cherry-Fimbrin	Gift from Tom Millard	N/A
D. melanogaster: UAS-GFP-Fascin	Zanet et al., 2009	N/A
D. melanogaster: UAS-GFP	Bloomington Drosophila stock center	RRID: BDSC_6874
D. melanogaster: UAS-GFP	Bloomington Drosophila stock center	RRID: BDSC_6658
D. melanogaster: UAS-myr-td-Tomato	Bloomington Drosophila stock center	RRID: BDSC_32221
D. melanogaster: UAS-rd-Tomato	Bloomington Drosophila stock center	RRID: BDSC_36327
D. melanogaster: UAS-nuclear-Red-Stinger	Barolo et al., 2004	N/A
D. melanogaster: UAS-GMA	Dutta et al., 2002	N/A
D. melanogaster: UAS-DN-Zipper-YFP	Dawes-Hoang et al., 2005	N/A
D. melanogaster: UAS-Reaper	Bloomington Drosophila stock center	RRID: BDSC_5824
D. melanogaster: srp>3xmCherry	Gyoergy et al., 2018	N/A
D. melanogaster: srp-GMA	Moreira et al., 2010	N/A
D. melanogaster: sqh>Sqh-GFP	Royou et al., 2004	N/A
D. melanogaster: Ubq>Hist-RFP	Bloomington Drosophila stock center	RRID: BDSC_23651
D. melanogaster: Ubq>RFP-α-tubulin	Basto et al., 2008	N/A
D. melanogaster: Ubq>GFP-α-tubulin	Basto et al., 2008	N/A
D. melanogaster: Attacin>GFP	Tzou et al., 2000	N/A
Software and Algorithms		
Volocity	PerkinElmer	http://cellularimaging.perkinelmer.com/downloads/
ImageJ/Fiji	Fiji	http://fiji.sc/
Photoshop	Adobe	http://www.adobe.com/uk/products/photoshop.html
Illustrator	Adobe	http://www.adobe.com/uk/products/illustrator.html
Prism	GraphPad	https://www.graphpad.com/scientific-software/prism/
Excel	Microsoft	https://www.microsoft.com/en-gb/
Other		
Glass bottom microscopy dish	Greiner Bio-One GmbH	627861
Multi-laser CLSM confocal microscope (Leica SP5II and SP8)	Leica	http://www.leica-microsystems.com/home/
63x NA1.4 Plan-Apochromat oil objective	Leica	http://www.leica-microsystems.com/home/
Zeiss Lightsheet Z.1 microscope	Carl Zeiss	https://www.zeiss.com/corporate/int/home.html

(*Continued on next page*)

Continued		
REAGENT or RESOURCE	SOURCE	IDENTIFIER
Perkin Elmer UltraView spinning disc system	PerkinElmer	https://science.nichd.nih.gov/confluence/display/mic/Perkin-Elmer+Ultraview+RS
Transmission electron microscope Tecnai 12-FEI 120kV BioTwin Spirit	Tecnai (Thermo Fisher Scientific)	https://www.fei.com/tecnai-upgrades/

CONTACT FOR REAGENT AND RESOURCE SHARING

Further information and requests for resources and reagents should be directed to and will be fulfilled by the Lead Contact Paul Martin (paul.martin@bristol.ac.uk).

EXPERIMENTAL MODEL AND SUBJECT DETAILS

Fly Stocks and Preparation

Drosophila melanogaster stocks were maintained on cornmeal molasses food in vials or bottles at 25°C and all crosses were performed at 25°C unless otherwise stated. For transgene induction using the Gal80^{ts} system, 0-1h old pre-pupae were shifted from 18 to 28°C for 16h prior to imaging at 25°C. The following fly lines were used in this study: w^{67} (as control), Lpp-Gal4 (from Pierre Leopold) and c564-Gal4 (Kambris et al., 2006) to drive transgene expression in fat body cells, srp-Gal4 (Brückner et al., 2004) to drive transgene expression in hemocytes and tub-Gal80ts (BL7017) to repress the UAS-Gal4 system in a temperature-dependent manner. The following UAS-lines were used: UAS-GFP-Ena (Gates et al., 2007), UAS-Cherry-Fimbrin (from Tom Millard), UAS-GFP-Fascin (Zanet et al., 2009), UAS-GFP (BL6874 and BL6658), UAS-myr-td-Tomato (BL32221), UAS-rd-Tomato (BL36327), UAS-nuclear-Red-Stinger (Barolo et al., 2004), UAS-GMA (Dutta et al., 2002), UAS-DN-Zipper-YFP (Dawes-Hoang et al., 2005) and UAS-Reaper (BL5824). Srp>3xmCherry (Gyoergy et al., 2018) was used to label hemocytes in a Gal4-independent manner and srp-GMA (Moreira et al., 2010) to label hemocytes and FBCs in a Gal4-independent manner. Sqh>Sqh-GFP (Royou et al., 2004), Ubq>Hist-RFP (BL23651), Ubq>RFP-α-tubulin (Basto et al., 2008) and Ubq>GFP-α-tubulin (Basto et al., 2008) were used to label the epithelium. Please note that these lines are not specific epithelial markers but also drive some expression in hemocytes, fat body cells and most other tissues. Attacin>GFP (Tzou et al., 2000) was used as a GFP reporter to show Attacin induction.

The genotypes of the pupae used in each experiment are summarised in the Table S1.

METHODS DETAILS

Microscopy and Wounding

Pupae were aged to the appropriate developmental stage (16-18h APF) in vials at 25°C by transferring newly formed white pre-pupae (0h APF) to the side of a fresh food vial using forceps and dissecting them after 16-18h. Pupae were placed on double-sided sticky tape on a glass slide and carefully removed from the pupal case with forceps before being placed on a glass bottom dish. Wounds were made using a nitrogen-pumped Micropoint ablation laser tuned to 435 nm (Andor Technologies, (Razzell et al., 2013)). For wound infection, overnight cultures of RFP-*E.coli* (Vlisidou et al., 2009) were washed 2x in PBS and resuspended 5x concentrated in PBS to OD 12.5. Wounded pupae were placed on a 1μl drop of RFP-*E.coli* for 5min and then imaged. Note that, since laser wounding only causes a 0.2-1μm wide hole in the cuticle, it is Pathogen-associated molecular patterns (PAMPs) rather than bacteria that enter this breach in the cuticle and act as activators at the wound site.

Most still images and movies were collected on a Leica TCS SP5 confocal microscope utilizing a 60x oil objective, except for Movie S1 which was generated on a Zeiss Lightsheet Z.1 microscope utilizing a 20x water immersion objective, or Movie S7 and Figure 4A where we used a Perkin Elmer UltraView spinning disc system with a 40x oil objective. Movies and images shown are maximum intensity projections made from approximately 30-60μm Z stacks or lateral views using the 3D-opacity mode in Volocity and were processed using ImageJ (NIH), Volocity (for Z-plane view), Adobe Photoshop or Adobe Illustrator software. If autofluorescence from the overlying cuticle hindered observation, the autofluorescence was manually erased before making maximum intensity projections. The heatmap of GFP fluorescence in Figures 3A and 3C and Movie S3 was made by setting the lookup table to "Fire" with ImageJ.

For transmission electron microscopy (TEM), pupae (16-17h APF) were removed from their pupal case, wounded and fixed in a mixture of 2% paraformaldehyde and 1.5% glutaraldehyde in 0.1 M cacodylate buffer (pH 7.4) and the same volume of heptane for 1h on a shaker, cutting part of the abdomen off after 30min for optimal penetration of fixative. After washes in 0.1 M cacodylate buffer (pH 7.4) and postfixation in 1% osmium tetroxide in 0.1M sodium cacodylate for 2h, the pupae were rinsed in buffer and in water, dehydrated in a graded ethanol series and embedded in Epon. The pupae were oriented to enable cutting of thick

transverse sections (1μm), which were stained with Methylene Blue for light-microscopic analysis. Thin sections (around 70 nm) were stained with uranyl acetate and lead citrate, and viewed by TEM (Tecnai 12 equipped with an FEI Eagle 4k x4k CCD camera).

Image Processing and Analysis

Wound front velocity was measured by subtracting the wound diameter at 30min from the wound diameter at 70min min, divided by 2 and reported in μm/h. Cell tracking was performed in NIH ImageJ (manual tracking) in 1min time intervals. FBCs that were recruited to the wounds were identified in the movies. The tracks of these wound-recruited FBCs, the FBCs that did not interact with the wounds, as well as all FBCs pooled, are depicted in Figure S2. In order to gather legitimate, comparable tracks from control, unwounded pupae for the measurements of the angle of migration, tracks of cells that did not pass through a circular area with a radius extending 25μm from the centre of the region of interest within the 30min time window were excluded from further analysis in Figure 2. The meandering index was calculated by dividing the distance that a cell travelled from its start point by the total track length. The angle of migration was measured between a line connecting the cell positions at 5 and 20min and the line connecting the cell position at 5min to the centre of the region of interest. Speed was calculated from the average of the velocities of 1min intervals from 5-10min post wounding.

QUANTIFICATION AND STATISTICAL ANALYSIS

Graphical representations and statistical analysis were generated in Prism (GraphPad) and Excel (Microsoft Office). Column scatterplots and line plots show the mean ± SEM of all the individual data from repeated experiments. Numbers of pupae/wounds analysed (n numbers) are shown in the figure legends. For statistical analyses, the data shown in column scatterplots were examined by the student's T-test.

Developmental Cell, Volume *44*

Supplemental Information

Fat Body Cells Are Motile and Actively Migrate to Wounds to Drive Repair and Prevent Infection

Anna Franz, Will Wood, and Paul Martin

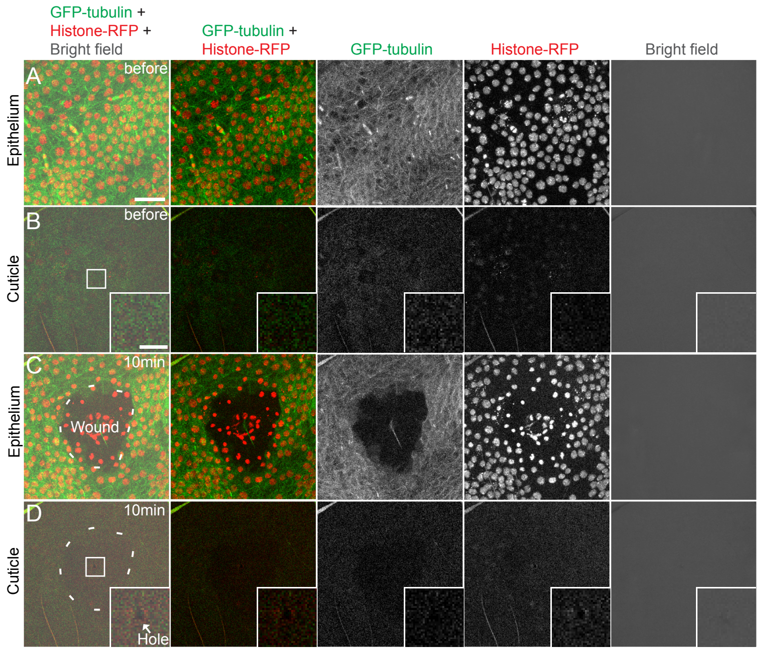

Figure S1
Morphology of a laser-induced epithelial wound labelled with Histone-RFP. Related to Figure 1

(A-D) Microscopy images of a Z-stack projection restricted to the epithelium (A, C) or the cuticle (B, D) before (A, B) and 10 minutes after (C, D) making a laser-induced wound (outlined by white dotted line) in a c564-Gal4+Ubi>GFP-tubulin+Ubq>Histone-RFP pupa (Tubulin in green; nuclei in red; bright field in grey; magnified in the insets to show the hole in the cuticle).
Scale bars=20μm and 5μm (insets).

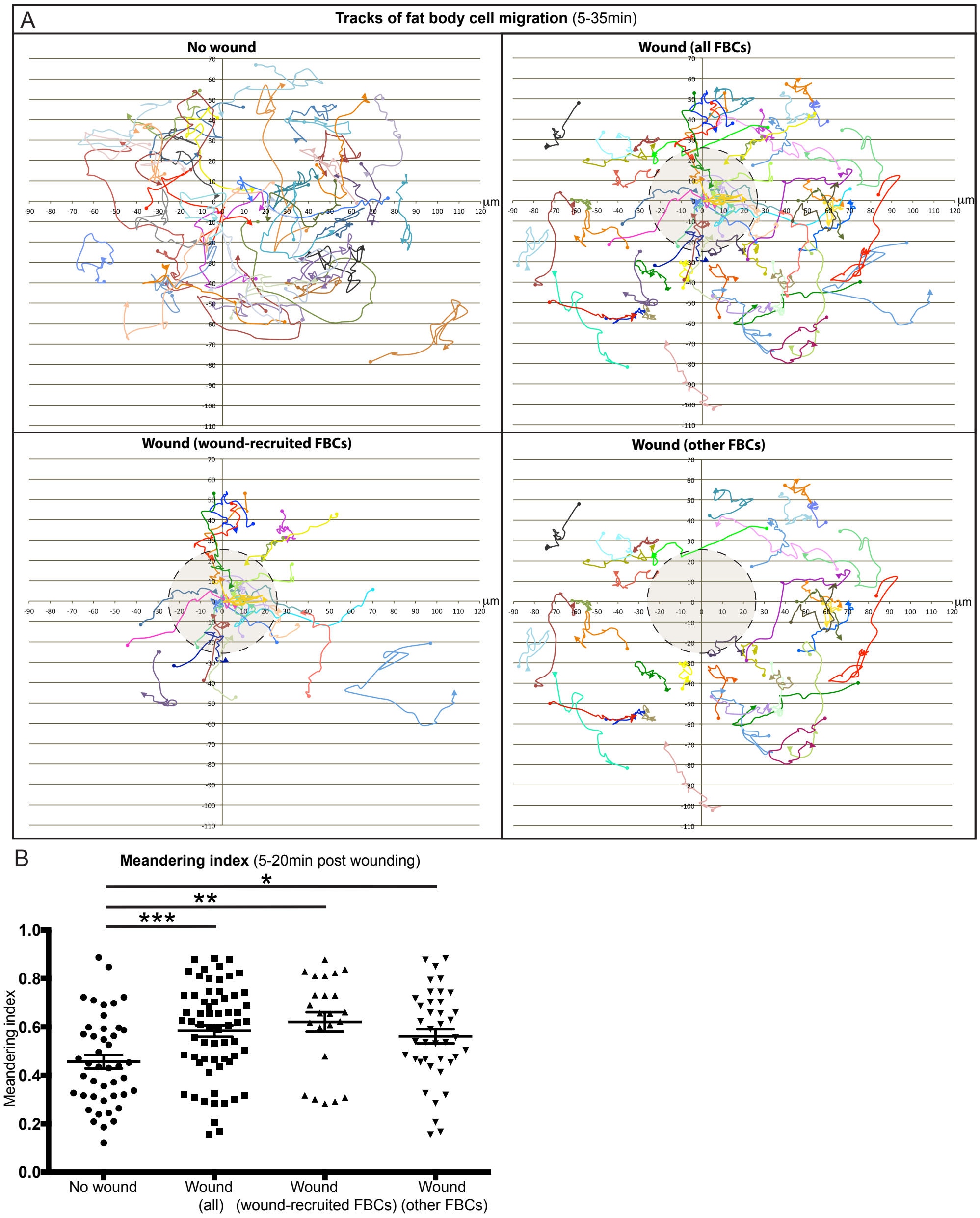

Figure S2

Fat body cells migrate to wounds with a high meandering index. Related to Figure 2

(A-B) Migration tracks of FBCs (A; 5-35min) and quantification of meandering index (B, 5-20min) of FBC migration in c564-Gal4+UAS-GFP+Ubq>Histone-RFP unwounded (n=44 cells) or wounded pupae (tracks from all FBCs displayed on top right; from wound-recruited FBCs displayed on bottom left; and from FBCs that do not to interact with the wound area, displayed on bottom right; n=63, 23 and 40 cells, respectively; tracking performed in Z-projection mode). Mean±SEM. * P<0.05, ** P<0.01, *** P<0.001 (Student's T-Test)

.

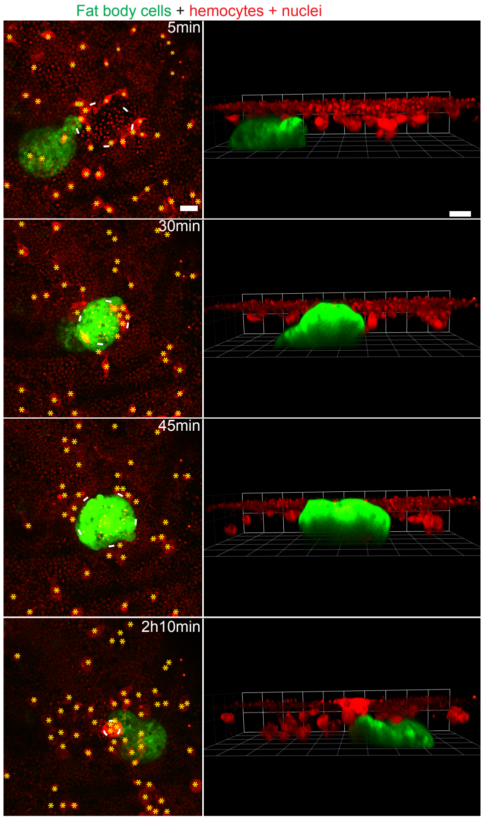

Figure S3
Migration of a fat body cell and hemocytes to a wound.
Related to Figure 4

Still images from a timelapse movie showing hemocyte and FBC recruitment to a wound in a c564-Gal4+UAS-GFP+srp>mCherry+Ubq>Histone-RFP pupa (projection (left) and Z-plane (right) views; FBCs in green; hemocytes in red with yellow asterisks; epithelial nuclei in red; wound area containing bright red nuclei + outlined with white dotted line). Elapsed time is in top right corner. Scale bars=20μm. See also Movie S8.

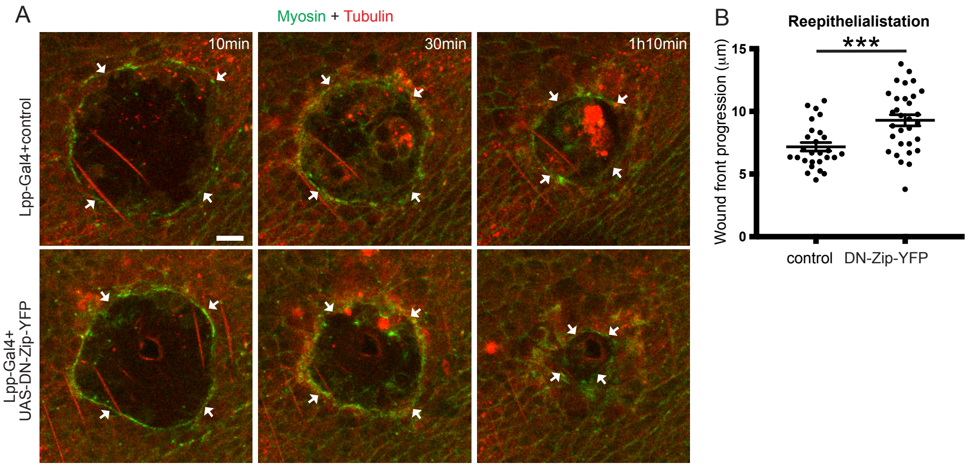

Figure S4
Reepithelialisation is faster in the absence of fat body cells at wounds. Related to Figure 3
(A-B) Still images from a timelapse of reepithelialisation (A) and quantification of wound front progression (30-70min, B) in sqh>Sqh-GFP+Ubq>RFP-tub+Lpp-Gal4+control or +UAS-DN-Zip-YFP pupae (n=27 and 31, respectively; Myosin in green; Tubulin in red; wounds margins indicated with arrows). Mean±SEM. ***P<0.001 (Student's T-Test). Control=w67. Scale bar = 10μm.

Table S1

Genotypes of pupae used in each experiment.

Related to Figures 1-5 and Supplementary Figures 1-4.

Figure	Genotype
Fig 1A	w67
Fig 1C-I	c564-Gal4+UAS-GFP+Ubq>Histone-RFP
Fig 2A-D	c564-Gal4+UAS-GFP+Ubq>Histone-RFP
Fig 3A and C	Lpp-Gal4+UAS-GMA
Fig 3D and E	Lpp-Gal4+UAS-rd-Tomato+w67 and<br>Lpp-Gal4+UAS-rd-Tomato+UAS-DN-Zip-YFP
Fig 4A	srp-Gal4+c564-Gal4+UAS-GFP+UAS-Red- Stinger
Fig 4B and C	srp-Gal4+UAS-Reaper+tubGal80ts+srp-GMA+Ubq>Histone-RFP
Fig 4D	c564-Gal4+UAS-GFP+Ubq>Histone-RFP
Fig 5A	c564-Gal4+UAS-GFP+Ubq>Histone-RFP
Fig 5B and C	w67
Fig 5D	c564-Gal4+UAS-GFP-Fascin+Ubq>RFP-tubulin
Figure 5 E	c564-Gal4+UAS-GFP+Ubq>Histone-RFP
Figure 5 F	Attacin>GFP+Lpp-Gal4+UAS-myr-td-Tomato
Fig S1A-D	c564-Gal4+Ubi>GFP-tubulin+Ubq>Histone-RFP
Fig S2A and B	c564-Gal4+UAS-GFP+Ubq>Histone-RFP
Fig S3	c564-Gal4+UAS-GFP+srp>mCherry+Ubq>Histone-RFP
Fig S4 A and B	sqh>Sqh-GFP+Ubq>RFP-tub+Lpp-Gal4+w67 and<br>sqh>Sqh-GFP+Ubq>RFP-tub+Lpp-Gal4+UAS-DN-Zip-YFP
